# Resection and reconstruction of a giant primitive neuroectodermal tumour of the abdominal wall with an ultra-long lateral circumflex femoral artery musculocutaneous flap: a case report

**DOI:** 10.1186/s12893-021-01095-5

**Published:** 2021-02-18

**Authors:** Xin Zhou, Pan You, Shuqing Huang, Xiang Li, Tongchun Mao, Anming Liu, Rongshuai Yan, Yiming Zhang, Wenlei Zhuo, Shaoliang Wang

**Affiliations:** 1 Department of Plastic and Cosmetic Surgery, Traditional Chinese Medicine Hospital, Southwest Medical University, Luzhou, Sichuan 646000 People’s Republic of China; 2 Department of Plastic and Cosmetic Surgery, Xinqiao Hospital, Army Medical University, Xinqiao Road, Sha Ping Ba District, Chongqing, 400037 People’s Republic of China; 3 Cancer Institute, Xinqiao Hospital, Army Medical University, Chongqing, 400037 People’s Republic of China

**Keywords:** Primitive neuroectodermal tumour, Musculocutaneous flap, Titanium polypropylene patch

## Abstract

**Background:**

Primitive neuroectodermal tumours are clinically rare. Here, we report a case of a large peripheral primitive neuroectodermal tumour of the abdominal wall. The defect was reconstructed with the longest lateral circumflex femoral artery musculocutaneous flap reported to date.

**Case presentation:**

A 15-year-old male suffered rupture and bleeding of an abdominal wall mass with a volume of approximately 23*18*10 cm^3^, involving the whole layer of the abdominal wall. Pathological examination revealed a peripheral primitive neuroectodermal tumour. The tumour was removed via oncologic resection, and the abdominal wall was reconstructed with a bilateral 44*8 cm^2^ lateral circumflex femoral artery musculocutaneous flap combined with a titanium polypropylene patch. The patient had smooth recovery postoperative, and the functions of the donor and recipient areas of the flap were not significantly affected.

**Conclusion:**

In this case report, we describe a rare primitive neuroectodermal tumour of the abdominal wall, which invaded almost the entire abdominal wall due to delay of treatment. After thoroughly removing the tumour, we immediately reconstructed the abdominal wall with an ultra-long lateral circumflex femoral artery musculocutaneous flap and achieved better appearance and function after the operation. This case suggests that we should adopt an integrated scheme of surgery combined with radiotherapy and chemotherapy in the treatment of peripheral primitive neuroectodermal tumours. Under the premise of determining the blood supply, the lateral circumflex femoral artery musculocutaneous flap can be cut to a sufficient length.

## Background

Primitive neuroectodermal tumours (PNETs) are a group of small round cell tumours originating from neuroectodermal cells; these tumours are rare and can be categorized as central PNETs (cPNETs) and peripheral PNETs (pPNETs) [1]. pPNET, which occurs mainly in young people, is a rare, highly invasive malignant tumour [2]. Although there is still no standard treatment, comprehensive treatment, the recommendation of which comprises extensive surgical resection combined with radiotherapy and chemotherapy, should be adopted.

In this case, the tumour was large and involved the entire abdominal wall. The repair of large area abdominal wall defects in regions with full thickness after complete resection of abdominal wall tumours has always been clinically difficult [3]. After tumour resection, we used a 44 × 8 cm^2^, super-long lateral circumflex femoral artery pedicled musculocutaneous flap for repair and reconstruction, and the effect was good. The report is as follows.

## Case presentation

**Fig. 1:**
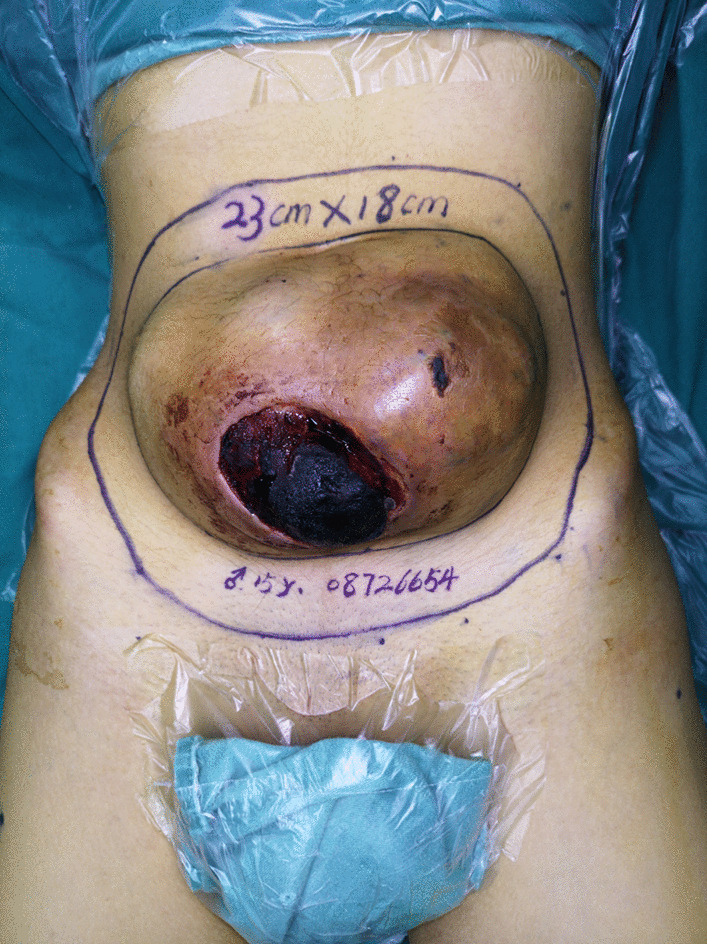
Local findings before surgery

The patient was a 15-year-old male who had no obvious adverse events related to a mass in the middle and lower abdominal wall a year ago. The mass was approximately the size of an egg and exhibited no obvious tenderness. The tumour was tightly adhered to the surrounding soft tissue. However, the tumour was neglected, and no medical treatment was administered. In the following year, the size of the mass increased progressively to a volume of approximately 23*18*10 cm^3^ (Fig. 1). While running at school, the patient suddenly felt abdominal pain and discomfort accompanied by bleeding from the abdominal wall mass. He was then admitted to plastic surgery. Upon examination at admission, abdominal CT revealed a soft tissue mass in the lower abdomen and anterior wall of the pelvis, the nature of which had yet been determined. Pathological biopsy revealed a small cell malignant tumour consistent with Ewing's sarcoma/pPNET [Desmin(−), EMA(−), S-100(−), TLE1(−), SATB2(−), Ki-6780%( +), CK(−), MyoD1(−), Vimentin( +), CD99( +), LCA(−), CK7(−), CD56( +), and NSE(−)].

After the patient was admitted, the tumour ruptured and began to bleed; therefore, immediate surgical resection of the tumour along with repair and reconstruction were required. In addition to active preparation of preoperative and postoperative care, the surgery was performed as follows. Along the edge of the mass, a 4-cm normal skin incision was made. The incision was performed subcutaneously into three layers of abdominal muscle and peritoneum; the tumour did not break through the peritoneum. To ensure that the tumour was removed completely, we removed the peritoneum. Tissues at the edge of the abdominal incision were obtained and sent for rapid pathological examination. The results showed that no diseased cancer cells were found, and the tumour was resected. First, the abdominal wall defect was covered with a 30*30 cm^2^ titanium polypropylene patch and then sutured along the edge of the incision to reconstruct the peritoneum. Then, a cloth sample was cut according to the size of the abdominal wall wound and divided into four parts along the longitudinal axis. The 44 × 8 cm^2^ flap was designed on both sides of the anterolateral thigh with twice the length and width of the cloth sample. The musculocutaneous flap was obtained with the line between the anterior superior iliac crest and the outer superior edge of the patella serving as the axis, and the ascending branch, transverse branch and descending branch of the lateral circumflex femoral artery served as the rotation point. The muscular branch of the femoral nerve accompanied by the lateral circumflex femoral artery was carried in the pedicled musculocutaneous flap to retain the innervation of the muscle flap.

The bilateral musculocutaneous flap was pedicled with the lateral circumflex femoral artery and vein. The subcutaneous passage was created in the bilateral inguinal area, and the musculocutaneous flap was guided to the abdomen through the rectus femoris muscle. The vascular pedicle was checked to ensure no torsion or compression, and the position of the two musculocutaneous flaps was adjusted as necessary. Two musculocutaneous flaps were cut into four musculocutaneous flaps: two vastus lateralis musculocutaneous flaps nourished by the descending branch of the lateral circumflex femoral artery and two tensor fascia lata musculocutaneous flaps nourished by the ascending and transverse branches of the lateral circumflex femoral artery. Two musculocutaneous flaps comprising the lateral thigh were placed on both sides of the abdominal wound, and two musculocutaneous flaps comprising the tensor fasciae lata were placed in the middle of the wound. Then, 3-0 tendon sutures were used to suture the distal and proximal abdominal muscle stump, and the broad fascia was sutured to strengthen the abdominal wall. A 4-0 silk suture was used to repair the abdominal wound with four pieces of spliced skin flap. The original navel position was reconstructed with a piece of full-thickness skin graft approximately 2 × 2 cm^2^ in size. The donor area of the thigh flap on both sides was sutured directly, and a drainage tube was placed. After 14 h of operation, we controlled the bleeding within 800 ml and finally completed the operation.

**Fig. 2:**
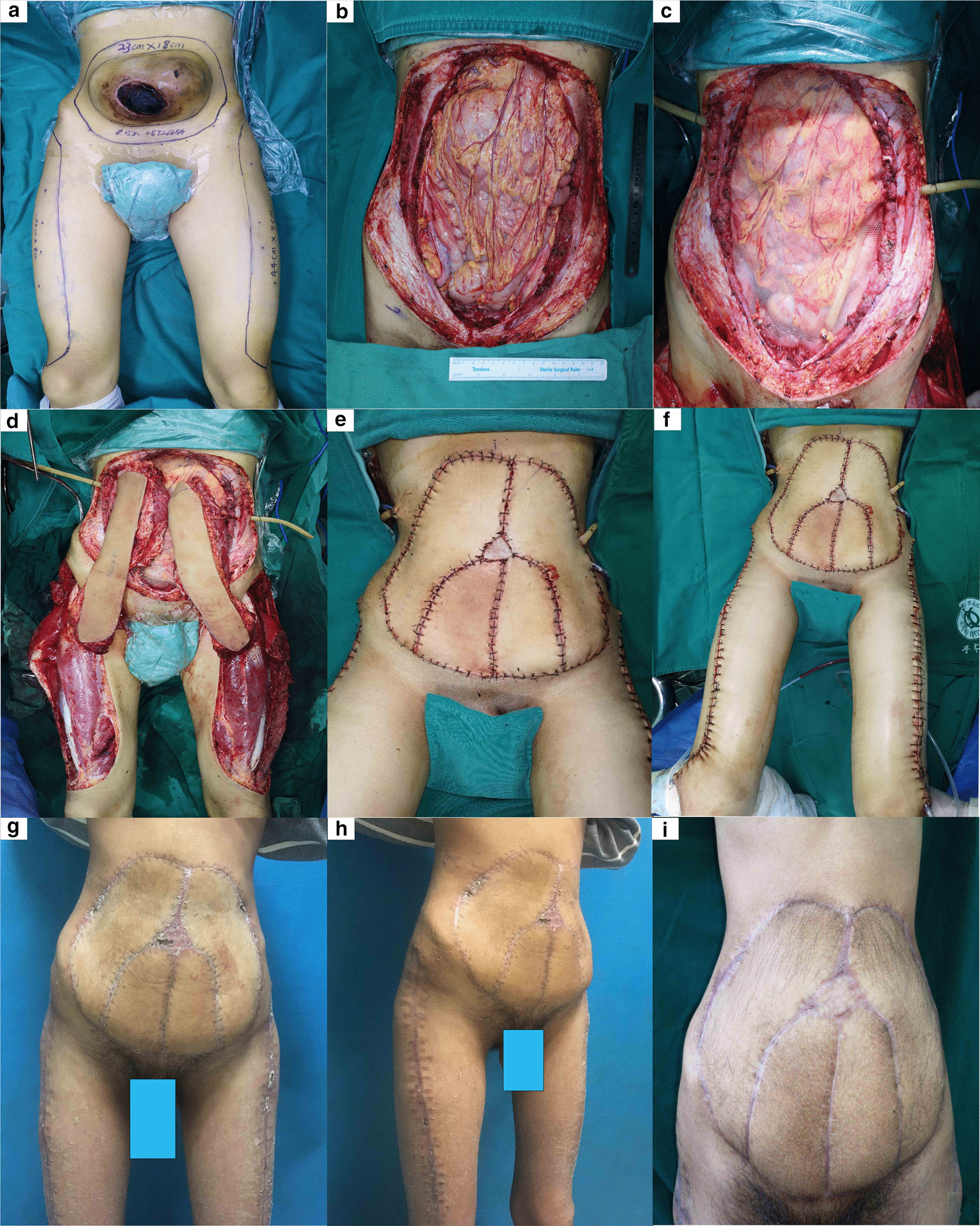
Preoperative, intraoperative and postoperative findings. **a** Findings of the tumour and the extent of the musculocutaneous flap. **b** Wound after complete resection of the tumour. **c** The abdominal wall defect was trimmed with titanium polypropylene patch material and then sutured along the edge of the incision to reconstruct the peritoneum. **d** The bilateral musculocutaneous flap was removed. **e** Findings of the abdominal wall after repair and reconstruction. **f** Findings of the supply area and the receiving area. **g** Posterior view 2 months after surgery. **h** Lateral view 2 months after surgery. **i** Positive view 6 months after surgery

**Fig. 3:**
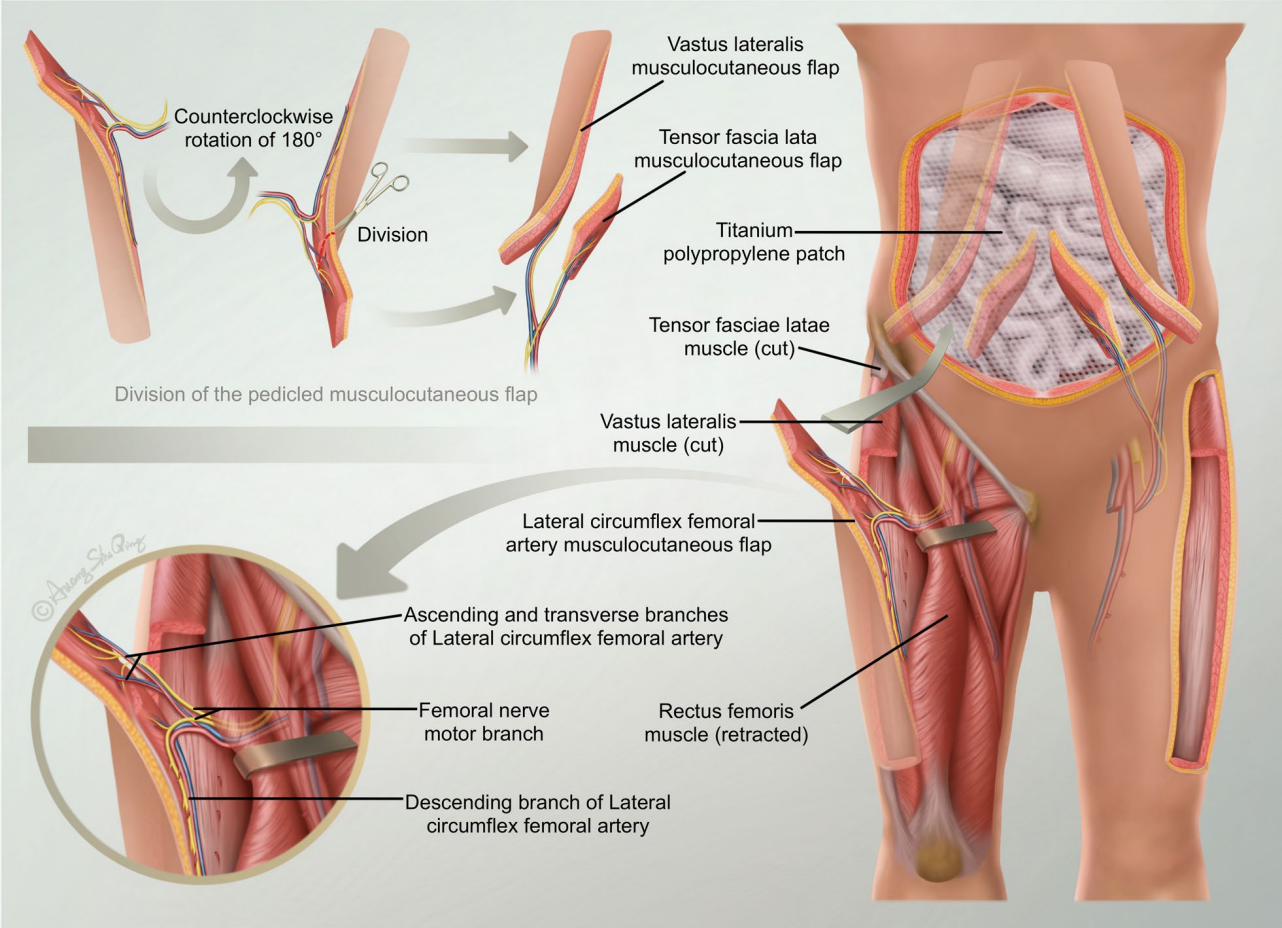
A diagrammatic summary of the surgical process

Postoperative symptomatic support treatment, such as adequate replenishment of blood volume, was provided according to the situation. The patient stayed on bed rest in the stoop and flexion position for 2 weeks to reduce vascular pedicle tension and avoid entrapment. After operation, the patient fasted until the gastrointestinal function returned to normal, and nutritional support was increased. Routine administration of antibiotics was conducted for 5 days to prevent infection. The stitches from the abdominal incision were removed on post-operative day 10, and the stitches from the thigh incision were removed on post-operative day 14. The wounds showed good healing and clean discharge. In the later stage of recovery, the patient was followed up for 3 months and recovered well. After operation, abdominal wall function was normal, and hernia and abdominal wall eminence were not found. No distant metastasis was observed, and the activity of the donor site of the musculocutaneous flap was not significantly affected (Figs. 2 and 3). The function of both lower limbs of the patient was normal, and squatting, standing and knee extension were not affected. The musculocutaneous flap transplantation also included the motor nerve of the lateral thigh muscle and the anterolateral thigh cutaneous nerve to ensure the movement and sensory function of the abdominal wall. Unfortunately, the patient refused radiotherapy and chemotherapy. Six months after the operation, the tumour developed a distant metastasis of the spine.

## Discussion and conclusions

With the gradual understanding of pPNET, the diagnosis and treatment of this disease are also improving, but its aetiology and origin remain unclear. pPNET occurs mainly in soft tissue, bone, the pelvis, the chest wall and the lungs [4]. Surgical resection combined with adjuvant radiotherapy and chemotherapy is the main treatment for pPNET [5]. The commonly used chemotherapy regimens include CAV (cyclophosphamide, doxorubicin, vincristine) and IE (etoposide, isocyclophosphamide) alternately for at least 12 cycles [6]. Radiotherapy is also used, especially for patients who cannot tolerate chemotherapy. Some pPNET patients undergo surgery after chemotherapy or radiotherapy but cannot receive adjuvant chemotherapy and radiotherapy given that combined therapy will increase the risk of complications [7].

In this case, the tumour involved the full thickness of the abdominal wall, and the volume was large. Thus, it was challenging to repair and reconstruct. Complete surgical resection is not difficult for surgeons, but repairing and reconstructing large abdominal wall defects after resection, which is the key to the success of the operation, remain an obstacle. For plastic surgeons, repairing large and complex abdominal wall defects is a clinical problem. At present, the methods that can be undertaken include direct suture [8] and implant material repair [9], the component separation technique (CST) [10], autologous tissue transplantation [11], abdominal wall dilatation [12], temporary abdominal closure [13] and combinations of these approaches. When considering the actual situation of the patient, we finally chose implant material repair combined with autologous tissue transplantation.

At present, there are two types of implant patch materials that can be used to repair abdominal wall defects: synthetic patches and biological patches. Synthetic patches include non-absorbable patches (such as polypropylene, polyester and expanded polytetrafluoroethylene, etc.), absorbable patches and composite patches, all of which can provide strong support for large abdominal wall defects after resection of abdominal wall lesions [14]. However, for intra-abdominal repair, given the issue of direct contact between the patch and abdominal organs, a composite patch with an anti-adhesion effect must be used; some examples include polypropylene-polytetrafluoroethylene composite patches and polypropylene-absorbable material patches for repair [15]. A comparative study of titanium polypropylene patches with other patches showed that the titanium polypropylene patch we used performed better in terms of serum swelling, postoperative pain, foreign body sensation, length of stay, and postoperative quality of life [16].

In the same area, the longest musculocutaneous flap was recorded as 36 cm [17]. The lateral circumflex femoral artery perforator flap is a common method to repair soft tissue defects. It can carry the tensor fasciae latae, aponeurosis and vastus lateralis muscle, which is useful not only to repair simple soft tissue defects but also to repair tendon defects and large deep hole wounds simultaneously [18]. Based on our experience, we believe the following: (1) The selection of tissue flaps should follow the principle of simplicity and practicality, and the sacrifice of healthy tissue should be reduced to a minimum. The pedicled tissue flap retains the blood supply of the tissue, and the outcome was satisfying or accepted. Its strong fascia and vascular pedicled tissue can resist intra-abdominal pressure. However, the location of the branch of the lateral circumflex femoral artery also determines the extent of the pedicled flap, and the free flap can be used if necessary. (2) The reliable blood supply allows the flap to have a large cutting area and has a certain advantage in repairing large area soft tissue defects in the abdominal wall. The degree of injury in the donor area should be considered in the design of the skin flap, and the width of the flap should be controlled within 10 cm to avoid long-term complications caused by skin grafting in the donor area. (3) The transfer tunnel in the inguinal area must also be expanded such that the skin flap can pass smoothly; this can shorten the length of the vascular pedicle to the maximum extent and avoid torsion and tension of the vascular pedicle. (4) When simultaneously filling the defect area of the rectus abdominis muscle flap, the direction of the muscle fibre should be consistent with the direction of the rectus abdominis muscle as long as possible. The function of the abdominal wall should be restored as much as possible. Attempts should be made to control the tension when anastomosing the stump of the vastus lateralis muscle and the rectus abdominis muscle so as not to oppress the nutritional vascular pedicle. (5) The lateral femoral motor nerve was wrapped in the vascular pedicle while part of the vastus lateralis muscle was simultaneously rotated to the abdomen. The skin flap with motor nerve preservation could prevent denervated muscle atrophy after surgery.

Although there has been considerable progress in the treatment of pPENTs, there is no unified treatment plan. At present, a comprehensive treatment scheme comprising surgical resection and radiotherapy and/or chemotherapy is advocated, but the effectiveness of these regimens needs further clinical verification. In addition, with the support of a reliable blood supply, the lateral femoral circumflex musculocutaneous flap can be cut long enough to ensure a sufficient amount of tissue to repair large tissue defects.

## Data Availability

The datasets used and/or analysed during the current study are available from the corresponding author on reasonable request.

## Abbreviations

PENTs: Primitive neuroectodermal tumours
cPNET: Central primitive neuroectodermal tumours
pPNETs: Peripheral primitive neuroectodermal tumours
